# Management of *de novo* nephrolithiasis after kidney transplantation: a comprehensive review from the European Renal Association CKD-MBD working group

**DOI:** 10.1093/ckj/sfae023

**Published:** 2024-02-06

**Authors:** Mehmet Kanbay, Sidar Copur, Cicek N Bakir, Alper Hatipoglu, Smeeta Sinha, Mathias Haarhaus

**Affiliations:** Department of Medicine, Nephrology, Koc University School of Medicine, Istanbul, Turkey; Department of Medicine, Koc University School of Medicine, Istanbul, Turkey; Department of Medicine, Koc University School of Medicine, Istanbul, Turkey; Department of Medicine, Koc University School of Medicine, Istanbul, Turkey; Department of Renal Medicine, Salford Royal NHS Institute, Northern Care Alliance NHS Foundation Trust, Salford, UK; Division of Renal Medicine, Department of Clinical Science, Intervention and Technology, Karolinska University Hospital, Karolinska Institutet, Stockholm, Sweden

**Keywords:** chronic kidney disease, crystal nephropathy, kidney stone disease, kidney transplant, nephrolithiasis

## Abstract

The lifetime incidence of kidney stones is 6%–12% in the general population. Nephrolithiasis is a known cause of acute and chronic kidney injury, mediated via obstructive uropathy or crystal-induced nephropathy, and several modifiable and non-modifiable genetic and lifestyle causes have been described. Evidence for epidemiology and management of nephrolithiasis after kidney transplantation is limited by a low number of publications, small study sizes and short observational periods. Denervation of the kidney and ureter graft greatly reduces symptomatology of kidney stones in transplant recipients, which may contribute to a considerable underdiagnosis. Thus, reported prevalence rates of 1%–2% after kidney transplantation and the lack of adverse effects on allograft function and survival should be interpreted with caution. In this narrative review we summarize current state-of-the-art knowledge regarding epidemiology, clinical presentation, diagnosis, prevention and therapy of nephrolithiasis after kidney transplantation, including management of asymptomatic stone disease in kidney donors. Our aim is to strengthen clinical nephrologists who treat kidney transplant recipients in informed decision-making regarding management of kidney stones. Available evidence, supporting both surgical and medical treatment and prevention of kidney stones, is presented and critically discussed. The specific anatomy of the transplanted kidney and urinary tract requires deviation from established interventional approaches for nephrolithiasis in native kidneys. Also, pharmacological and lifestyle changes may need adaptation to the specific situation of kidney transplant recipients. Finally, we point out current knowledge gaps and the need for additional evidence from future studies.

## INTRODUCTION

Nephrolithiasis is a global health problem with a lifetime risk of 6%–12% in the general adult population, which has increased considerably over recent decades, in tandem with an increase in the prevalence of metabolic syndrome [1]. Despite the localized manifestation of the disease in the urinary tract, nephrolithiasis is lately being recognized as a systemic disorder strongly associated with chronic kidney disease (CKD), bone mineral disorders, hyperparathyroidism, metabolic syndrome, coronary artery disease, type II diabetes mellitus and hypertension [2]. Multiple non-modifiable (i.e. family history, genetic polymorphisms, past medical history) and modifiable risk factors (i.e. dietary intake and urinary excretion of various substances) have been identified [3, 4]. Nephrolithiasis can cause acute kidney injury (AKI) and CKD, and may even lead to end-stage kidney disease (ESKD) through obstructive nephropathy or crystalline-induced kidney injury.

**Figure 1:**
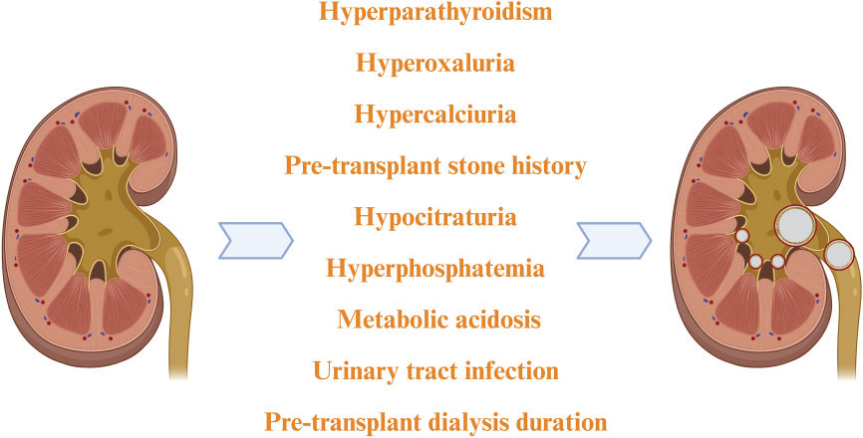
Possible risk factors for post-transplant nephrolithiasis.

*De novo* nephrolithiasis after kidney transplantation can potentially threaten kidney graft function and survival. As nephrolithiasis is strongly linked to CKD and recognized as a chronic condition with up to 50% recurrence rate when left untreated, there is growing interest in diagnosis and management of kidney stones after renal transplantation [5]. Female gender, a history of kidney stone disease before transplantation, gout, hyperparathyroidism, hypertension, hypercalcemia and hyperphosphatemia, a longer pre-transplant dialysis vintage, urinary stasis or urinary tract obstruction, and urinary tract infections (UTI) have been identified as risk factors for nephrolithiasis in the transplanted kidney (Fig. 1) [6–9]. Post-transplant nephrolithiasis was first described by Hume and colleagues in 1966, enhancing clinical awareness of the condition [10]. In this narrative review, our aim is to describe the prevalence, risk factors, outcomes and therapeutic approaches of *de novo* nephrolithiasis in renal allografts among kidney transplant recipients along with the evaluation of such condition as an aetiological factor for AKI, CKD and/or ESKD.

## NEPHROLITHIASIS AS A CAUSE FOR KIDNEY INJURY

### Acute kidney injury

Nephrolithiasis is a rare cause of AKI, accounting for only 1%–2% of AKI cases in adults, although it is more common in children [11]. Nephrolithiasis is one of the post-renal aetiologies of AKI; when it obstructs the urinary flow, the increased tubular pressure transfers to Bowman's space and decreases glomerular filtration rate (GFR) [12]. Following obstruction, vasoactive mechanisms, including the renin–angiotensin system (RAS), prostaglandins, kinin–kallikrein system and thromboxane, become activated. This activation leads to vasoconstriction, exacerbating the decline in the GFR and contributing to ischaemic injury [13]. As early as 4 h after the hydrostatic changes, the inflammatory response becomes evident by macrophage infiltration, followed by neutrophils and T cells [14]. Ischaemic injury and RAS activation induce oxidative stress, triggering various inflammatory pathways, including nuclear factor-κB and transforming growth factor-β, which increase inflammatory cytokines and contribute to kidney injury [14]. Besides obstruction of the urinary tract, supersaturation of solutes in the glomerular ultrafiltrate can cause tubular crystalline nephropathy [15]. The supersaturation can arise due to factors such as dehydration, over-excretion of calcium and oxalate, alteration in urine pH and certain medications [11]. Although the underlying pathophysiology is not completely known, crystals can obstruct kidney tubules and cause direct cytotoxicity or indirect toxicity by inducing inflammation and cellular necrosis [15]. In contrast to nephrolithiasis, which presents with flank pain, patients typically exhibit no overt symptoms and present solely with an elevation in serum creatinine levels [16].

### Chronic kidney disease

Epidemiological studies indicate an association between nephrolithiasis, CKD and ESKD [17–20], independent of geographical factors or CKD stage [21]. The risk of ESKD seems to be increased in recurrent symptomatic and asymptomatic, compared with incident, stone formers [22]. Compared with other stone formers, uric acid stones seem to be more often associated with higher neutrophil to lymphocyte ratio and CKD risk [23]. The fact that the risk of developing CKD persists even after adjustment for age, sex and metabolic confounders (hypertension, diabetes, hyperlipidaemia and cardiovascular disease) suggests that there might be a direct causal relationship between nephrolithiasis and CKD [24]. Nephrolithiasis also increases the risk of UTI, contributing to the increased risk of CKD [25–27].

## DISTURBED URINARY SOLUTE EXCRETION—PREDESTINATION FOR NEPHROLITHIASIS AND CKD

### Hyperoxaluria

Hyperoxaluria increases the risk of nephrolithiasis. It exists as an inherited condition due to hepatic overproduction of oxalate, also referred to as primary hyperoxaluria [28, 29], or secondary hyperoxaluria in response to an increase in oxalate or oxalate precursor intake (e.g. ascorbic acid, certain nuts, tea and fruits) or a decline in intestinal oxalate metabolism or fat malabsorption, e.g. in response to surgery (i.e. Roux-en-Y gastric bypass or small bowel resection), intestinal disorders (i.e. celiac disease, Crohn's disease), pancreatic disorders (i.e. chronic pancreatitis, exocrine pancreatic insufficiency) or drugs (i.e. orlistat) [30]. Clinical outcomes, including the formation of calcium oxalate stones in the urinary tract or crystal formation in the renal parenchyma, termed as oxalate nephropathy, are similar in both types of hyperoxaluria. In addition to the formation and deposition of calcium oxalate stones, excess oxalate also impairs the proliferation of renal epithelial cells, induces apoptosis and pro-fibrotic signals, stimulates epithelial-to-mesenchymal transformation [31, 32], induces oxidative stress [33], and promotes pro-inflammatory cells and the NLRP3 inflammasome [34, 35].

Hyperoxaluria can cause progressive kidney injury and oxalate crystal deposition in other organs due to decreasing renal excretion with declining GFR [28]. Up to 50% of patients with primary hyperoxaluria are diagnosed at advanced stages of CKD and up to 10% of patients are diagnosed after recurrence of nephrolithiasis following kidney transplantation [36]. As untreated primary hyperoxaluria almost certainly recurs after kidney transplantation, early diagnosis is of great importance.

### Hyperuricosuria

Uric acid stones constitute approximately 5%–40% of all kidney stones [37]. While some rare congenital forms exist, the most common cause is idiopathic hyperuricosuria associated with diabetes mellitus, obesity and the metabolic syndrome [38]. Increased uric acid excretion can be induced by gout and a purine-rich diet, chronic diarrhoea, cancer with high cell turnover and uricosuric medications. Reduced hepatic ammonia production and an acidic urine facilitates uric acid stone precipitation. Hyperuricaemia and uricosuria increase the risk of progressive CKD. In addition to renal outflow obstruction, mechanisms of kidney damage include the induction of renal vasoconstriction and impaired autoregulation, inflammation and microvascular damage [39]. The effect on CKD progression may be more prominent in early than in late CKD stages [40]. Uric acid stones are among the most common causes of *de novo* nephrolithiasis in kidney transplant recipients; thus, early identification is of importance for prevention of progressive renal graft damage [41].

### Hypocitraturia

Urinary citrate excretion has an inhibitory effect on nephrolithiasis. Hypocitraturia is estimated to be the pathogenic factor in approximately 30% (10%–60%) of all kidney stone formers [42]. Starvation, bariatric surgery, use of proton pump inhibitors, testosterone, hypoparathyroidism or any type of acidosis reduce urinary citrate excretion, while female sex, oestrogens, metabolic alkalosis, hyperparathyroidism, vitamin D and growth hormone increase urinary citrate concentrations [43]. Citrate can complex calcium ions and increase their solubility. Thus, kidney stones in hypocitraturia are mostly calcium-containing stones. Hypocitraturia is more prominent after renal transplantation and predisposes for post-transplant nephrolithiasis [44].

## NEPHROLITHIASIS AMONG KIDNEY TRANSPLANT RECIPIENTS

### Prevalence

A retrospective cohort study involving 42 096 kidney transplant recipients between 1994 and 1998 in the USA revealed a low hospitalization rate for kidney stones (104 cases/100.000 person-years). However, this study was limited by a short period of follow-up (mean 1.89 years) and inclusion of only hospitalized cases of nephrolithiasis [8]. A more recent study, utilizing the United States Renal Data System and involving 83 535 renal transplant recipients between 2007 and 2018, has demonstrated a prevalence of kidney stone disease of 1.7% within 3 years after transplantation with a median time from transplantation to stone disease of 0.61 years [25–75, confidence interval (CI) 0.19–1.46 years] [9]. Several retrospective single-centre studies with longer follow-up time demonstrated nephrolithiasis rates of 0.46%–1.29% after kidney transplantation [45–49].

A large-scale systematic review and meta-analysis conducted in 2016 evaluated the prevalence and characteristics of nephrolithiasis among kidney transplant recipients in 21 clinical trials [7]. The estimated incidence of nephrolithiasis was 1% (95% CI 0.6%–1.4%) with diagnosis made on average 28 ± 22 months after transplantation among a total of 64 414 kidney transplant recipients. The heterogeneity of incidence rates was high and many included studies did not report total follow-up time. Calcium-based stones comprised the largest percentile (67%) of nephrolithiasis cases (30% mixed calcium oxalate-calcium phosphate, 27% calcium oxalate and 10% calcium phosphate) followed by struvite (20%) and uric acid stones (13%). Risk factors for kidney stone development included hypercalciuria, hyperparathyroidism, hypophosphatemia, hypocitraturia, UTI or obstruction [41].

To conclude, prevalence of nephrolithiasis among kidney transplant recipients is approximately 1%–2%, which is lower than in the general population [1], but the existing evidence is limited by heterogeneity of results and short follow-up periods in most studies involving transplant recipients. Moreover, kidney stone disease has considerable variations in terms of prevalence depending on age, gender and ethnicity. Thus, generalizability of findings regarding the low rates of nephrolithiasis among transplant recipients may be misleading and should be interpreted with caution.

### Clinical presentation

The clinical presentation of nephrolithiasis in transplant recipients may differ from the classical presentation [50]. Since renal transplant recipients require frequent monitoring, including imaging of the renal graft, asymptomatic kidney stones may be more frequently detected than in the general population [51]. On the other hand, pain may be less prevalent because of denervation of the transplanted kidney, leading to later diagnosis and more frequent complications, such as hydronephrosis and AKI [52]. Boissier *et al*. [53] demonstrated that diagnosis was incidental in 34% of kidney transplant recipients with nephrolithiasis, while 17% of the patients presented with a rise in creatinine, 10% with gross hematuria, 9% with urinary tract infection and only 3% with pain. The mean (minimum–maximum) age at diagnosis was 44 (11–72) years, and the mean time interval from transplant to nephrolithiasis was 28 (3–387) months [53]. Due to denervation of the transplanted kidney, the rate of asymptomatic cases may be higher in transplant recipients than in the general population. The insidious clinical features should raise the index of suspicion of nephrolithiasis in kidney transplant recipients. Atypical presentations should also lower the threshold for imaging.

### Risk factors

Studies investigating the potential risk factors for kidney stone disease among kidney transplant recipients are scarce. As potential risk factors are not clearly identified, preventive measures may not be initiated in time or at all. Ganesan *et al*. showed that a pre-transplant history of nephrolithiasis has the highest hazard ratio for *de novo* post-transplant disease in the kidney graft [9]. In addition, persistence of risk factors including hyperoxaluria, hypercalciuria, hypocitraturia, hyperphosphatemia, inadequate management of hyperparathyroidism and urinary tract infections are the main aetiological factors for nephrolithiasis in the kidney allograft after transplantation (Fig. 1). Another rare cause for nephrolithiasis among kidney transplant recipients are the ‘forgotten’ ureteral stents which aim to decrease major urological complications and recommended for all transplant recipients in multiple reports [54]. A prospective clinical study involving 68 kidney transplant patients with a mean follow-up period of 17 months has demonstrated poor clinical consequences of forgotten ureteric stents in terms of renal functions and nephrolithiasis [55].

**Figure 2:**
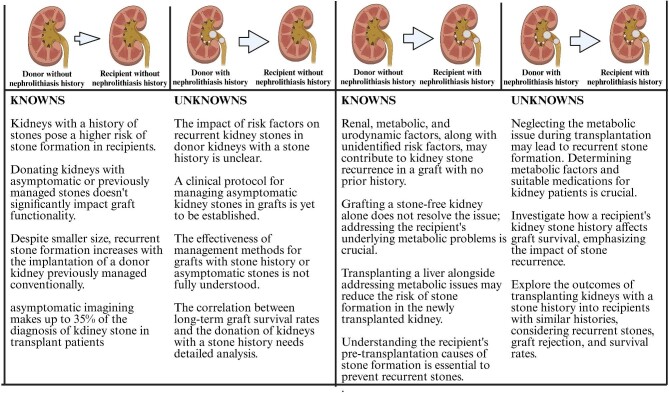
Knowns and unknowns about the relationship between kidney stone history in recipient and/or donor and approach to different situations up-to-date.

Figure 2 outlines our current understanding of the relationship between the kidney stone history in the recipient and/or donor. However, in a recent meta-analysis, only 6% of renal transplant patients with nephrolithiasis had a history of kidney stones before transplantation [56].

## URINARY SOLUTE CONCENTRATIONS AFTER KIDNEY TRANSPLANTATION

A crucial risk factor for kidney stone disease is the urinary excretion of various solutes with stone forming potential. Among these, calcium and oxalate are important stone forming solutes, whereas citrate can have a protective effect. Urinary super-saturation with calcium and oxalate or low levels of urinary citrate are identified as risk factors for kidney stone disease in the general adult population, and hypocitraturia and hyperoxaluria are even more prominent in renal transplant recipients [44, 57, 58]. A comparative study demonstrated supersaturation of calcium oxalate, octacalcium phosphate and brushite salts in healthy subjects, while concentrations were markedly lower despite higher oxaluria and lower citraturia in renal transplant recipients, who at the same time demonstrated lower calciuria and higher urine volume [58]. When comparing individuals with similar urine volumes, only brushite and octacalcium phosphate saturations remained lower in renal transplant recipients, while there was no difference in apatite and calcium oxalate saturations. Another study demonstrated that in the presence of normocalciuria, hypocitraturia and hyperoxaluria are the major risk factors for kidney stone disease [44]. Since hypocalciuria and an increased urine volume are relatively common in kidney transplant recipients, this may explain the lower observed rates of nephrolithiasis after renal transplantation.

The underlying pathophysiological mechanism leading to hypocitraturia is unclear, with several potential hypothetical mechanisms; kidney transplant recipients are prone to metabolic acidosis due to allograft function and medications along with renal tubular acidification defects related to calcineurin inhibitor therapy, can cause intracellular acidosis in proximal tubules, which enhances citrate reabsorption [59, 60].

## DIARRHOEA

Another contributing factor may be an increased prevalence of diarrhoea, due to infectious diseases, side effects of mycophenolate mofetil treatment or frequent antibiotic exposure, leading to a decline in intestinal citrate absorption [61]. Diarrhoea and intestinal malabsorption may also impair the absorption of magnesium, leading to lower urinary magnesium content, and enhance the absorption of oxalate [62]. Low urinary magnesium content has been identified as a potential risk factor for nephrolithiasis, as magnesium can inhibit the nucleation and growth of calcium-oxalate stones and increase urinary citrate concentrations through chelation of citrate in urine preventing tubular reabsorption [7].

## CHRONIC KIDNEY DISEASE–MINERAL BONE DISORDER

While nephrolithiasis is uncommon in secondary hyperparathyroidism, treatment with vitamin D and calcium supplementation have been identified as potential risk factors for hypercalciuria, nephrocalcinosis and nephrolithiasis. A meta-analysis study involving 451 patients with nephrolithiasis and 482 control patients from six case–control studies and one randomized control trial has demonstrated statistically significant association between serum vitamin D levels and nephrolithiasis risk [63]. Similarly, the link between higher 1,25-hydroxy vitamin D levels and symptomatic kidney stones has been established in another study with a 12-year follow-up period [64]. However, contradictory findings have also been described in the literature [65, 66]. On the other hand, calcimimetic agents, mainly cinacalcet, appear to be safe in terms of nephrocalcinosis and nephrolithiasis [67]. Persistent hyperparathyroidism after kidney transplantation has been associated with renal calculus formation [7], nephrocalcinosis and reduced kidney graft function [68].

### Outcomes

An important aspect of kidney stone disease in kidney transplant recipients is the impact on allograft function and survival, considering the association of nephrolithiasis with unfavourable renal outcomes in the general population. It is thus surprising that evidence is limited linking kidney stones to graft function and survival after kidney transplantation. A small number of case reports have been published illustrating cases of obstructive nephropathy and kidney graft failure due to renal or ureteral calculi [69–71]. A single-centre retrospective observational study conducted in 574 kidney transplant recipients, with a mean (±standard deviation) follow-up period of 55 ± 53 months and nephrolithiasis prevalence of 4.4%, demonstrated that nephrolithiasis did not have an impact on allograft survival (odds ratio 1.04, CI 0.708–1.54, *P *= .824) [72]. Similarly, no negative impact of kidney stone disease on graft function has been detected, either at the time of kidney stone diagnosis or after stone removal, in a retrospective single-centred observational study including 849 transplant recipients with 1.8% prevalence rate and mean follow-up period of 58 months [50]. Another single-centre study evaluating the management of asymptomatic donor-derived ≤4 mm stones left *in situ* during transplantation included 31 patients with a mean (minimum–maximum) stone size of 2.9 mm (1–4.3 mm). During a mean follow-up of 43.1 months, 83.9% of the patients experienced spontaneous passage of the stone as evaluated via computed tomography, irrespective of the location within kidney, while 6.4% patients continued without symptoms. Nevertheless, three patients (9.6%) required surgical intervention due to the development of symptoms or complications. This study demonstrates the benign nature of small asymptomatic kidney stones after kidney transplantation with high rates of spontaneous passage, eliminating the need for surgical intervention [73]; this is supported by a further study evaluating small kidney stones after renal transplantation [74].

Even though these findings suggest that post-transplant nephrolithiasis may not affect allograft function or survival, the limited evidence does not support any solid conclusions regarding the long-term risk for kidney transplant recipients. Based on the experience in the general population, we advocate to have a high degree of suspicion and to carefully monitor transplanted patients with nephrolithiasis.

### Management

Treatment of kidney stone disease has evolved over the years, thanks to the technical refinement of endoscopes and surgical methods, laser technology and diversification of auxiliary equipment. Open surgery has largely been replaced by minimally invasive treatment alternatives such as shock wave lithotripsy (SWL), semi-rigid ureteroscopy (URS), retrograde intrarenal surgery (RIRS), percutaneous nephrolithotomy (PNL) and endoscopic combined intrarenal surgery. The choice of therapeutic approach, including also conservative management, depends on the clinical presentation, aetiology, size, number and location of the stone [75].

Since kidney stones in renal transplant patients often are asymptomatic and patients have a solitary functioning kidney, they pose a risk for deteriorating renal graft function, particularly if urinary outflow obstruction is present. Moreover, due to the shortened urinary tract and an increased susceptibility to infections in kidney transplant recipients, nephrolithiasis may cause complicated UTI and urosepsis. In such cases, the collecting system must be immediately decompressed by percutaneous nephrostomy catheter insertion or ureteric stent placement along with antibiotics which may postpone the definitive therapeutic intervention [64].

A challenge in post-transplant nephrolithiasis management is the altered anatomical location of the allograft and varying ureterovesical and pyelo-ureteral anastomoses. When deciding the optimal therapeutic modality in transplant recipients, the size, number and location of the stones should be considered. As small stones (<4 mm) are likely to pass spontaneously, conservative management with close follow-up is usually the treatment of choice [76]. However, this approach demands meticulous clinical, radiologic and laboratory monitoring, therefore watchful waiting is not frequently utilized [77]. Since residual stones and recurrence of nephrolithiasis are frequently reported in the literature, choosing the most efficient intervention, and correcting the predisposing factors, including metabolic abnormalities, avoiding UTI, and using metaphylaxis for nephrolithiasis are essential [51].

**Figure 3:**
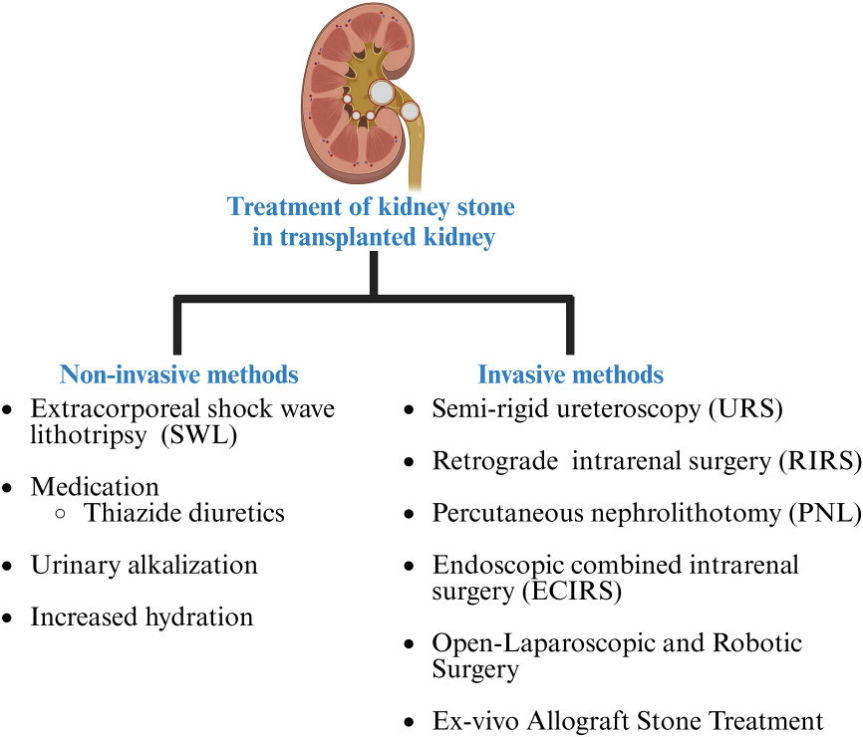
Diverse treatment modalities for post-transplant nephrolithiasis.

A recent meta-analysis of interventions in transplanted kidneys reported stone-free rates (SFR) at 3 months as 96% with open surgery, 95% with antegrade ureteroscopy, 86% with PNL, 81% with retrograde ureteroscopy, 75% with SWL and 62% with medical treatment. Overall, 52% of the patients required a surgical intervention including open surgery (4%), ureteroscopy (29%) and PNL (19%), which is higher than in patients with native kidneys [53, 78]. Although the most common treatments include retrograde URS and SWL for large stones, these findings suggest that anterograde approaches such as antegrade ureteroscopy, percutaneous nephrolithotomy and open surgery are more effective and may be considered more in allograft nephrolithiasis than in native kidneys. In Fig. 3 different management options are shown and in the following sections, we summarize these methods.

#### Conservative management

Conservative management is the preferred approach for non-obstructive stones (<4 mm) [56, 79–81] and includes strategies for pain control, medical expulsive therapy and therapies to prevent recurrence. Non-steroidal anti-inflammatory drugs, the treatment of choice in the general population, are not recommended in kidney transplant recipients due to their potential nephrotoxicity [82]. Alpha-blocker agents are the initial choice for medical expulsive therapy in the general population and for transplant recipients [82, 83]. Urinary alkalinization and increased hydration can cause complete resolution of uric acid stones [79, 84]. However, conservative management has a low SFR, and laboratory, as well as radiological, follow-up is highly recommended [53, 76].

**Figure 4:**
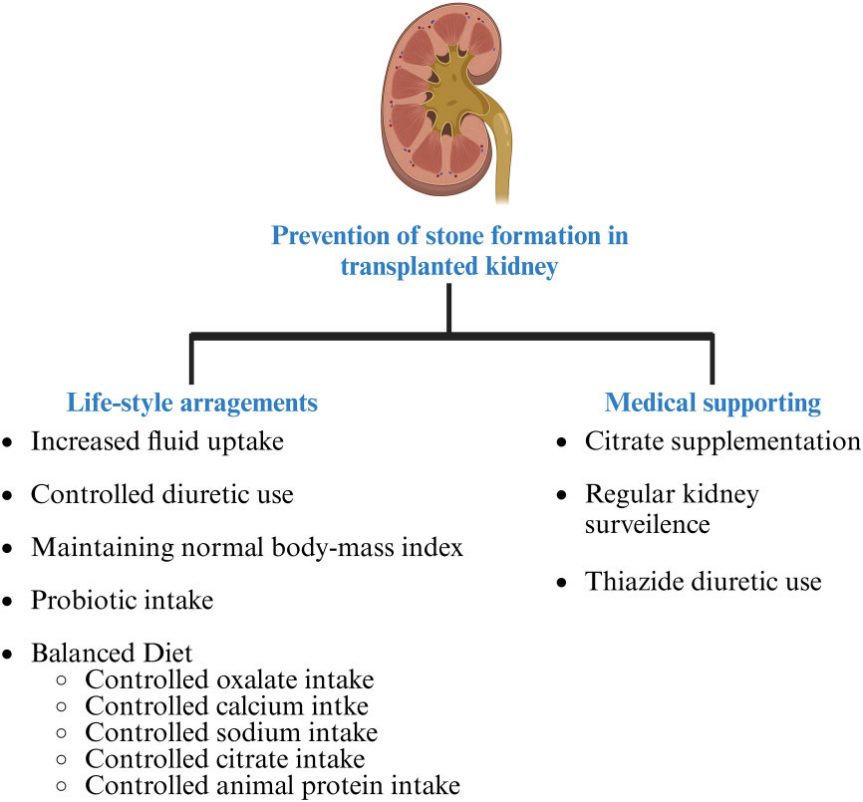
Recommended prevention methods for stone formation.

General recommendations for lifestyle modifications include fluid intake of 2–2.5 L/day to achieve urine specific gravity at or below 1.010 along with a balanced diet are the recommended for prevention [85, 86]. In addition, thiazide diuretics, citrate supplementation, ideally potassium citrate, and allopurinol are recommended for patients with recurrent nephrolithiasis and other methods showed in Fig. 4 as prevention methods of stone formation in general population [87]. Even though the known protection capability of these methods against stone formation is apparent, the applicability of these recommendations to transplant recipients is unclear pending evidence from large-scale studies.

#### Extracorporeal shock wave lithotripsy

Extracorporeal shock wave lithotripsy is the most commonly employed minimally invasive method utilized in the management of nephrolithiasis with low rates of complications [88]. Primary indications include small-to-medium sized stones (<1.5 cm), located at the ureter and kidney, except for the lower pole. Two systematic reviews investigating SWL in transplant kidneys reported SFRs of 75% at 3 months [59] and 80% at varying follow-up times [89]. Although SWL is minimally invasive, the anatomical position of the graft kidney close to the iliac bone could attenuate the shock waves and decrease the effectiveness of the procedure [47, 52, 90]. Since low-voltage SWL is preferred to minimize the effect on renal allografts, multiple treatment sessions are frequently needed, which creates a further healthcare burden [76, 91]. Furthermore, the graft may retain residual stone debris, which has the potential to cause asymptomatic ureteral obstruction, making it crucial to maintain close monitoring after SWL treatment [92].

#### Percutaneous nephrolithotomy

Percutaneous nephrolithotomy is the treatment of choice for large stones (>2 cm) [93, 94] and its use after kidney transplantation was initially described by Hulbert *et al*. [95]. The superficial location of the graft kidney facilitates PNL, however, it also predisposes to bowel injury. Besides, the immunosuppressants can cause perinephric fibrosis that may impede the procedure and increase complications [96]. Nevertheless, the literature reports high SFR and minimal complications for PNL in transplant kidneys [94].

#### Antegrade and retrograde ureteroscopy

Antegrade and retrograde ureteroscopy with a semi-rigid ureteroscope is the method of choice with low complication rates for stones located in the distal to mid-ureter, whereas RIRS using a flexible ureteroscope is the preferred option for proximal uretra and kidney stones [77]—additionally, it is preferred among pregnant or obese patients or patients with bleeding diathesis [97]. For smaller stones in the transplant kidney, an endoscopic approach is also favoured [98]. While the retrograde approach (transurethral) is more commonly employed, the antegrade approach (percutaneous access) yields a higher SFR [53]. Despite the complex anatomy of the transplanted kidney, the development of thinner, flexible ureteroscopes and laser technology makes ureteroscopy a valid therapeutic option for calculi in transplant recipients. However, UTI is a known complication that should be considered [79].

#### Open-laparoscopic and robotic surgery

In the era of minimally invasive treatment, open stone surgery is reserved only for complex cases, including when concurrent procedures are also indicated (e.g. treatment of ureteral stenosis) or when minimally invasive treatment has failed [99]. Although the use of immunosuppressants and the anatomy of the transplanted kidney close to the iliac vessels make open surgery challenging, it has a higher SFR compared with other interventions [53, 75]. Although no data about the outcomes of laparoscopic and robotic surgery in transplanted patients are available in the literature so far, they have been successfully performed for stones in the pelvic ectopic kidney [100–102], indicating a potential use also in transplanted kidneys.

#### Ex vivo allograft stone treatment

Around 5% of asymptomatic donors are estimated to have small (<15 mm), non-obstructing stones in the urinary tract of the graft kidney [103], which are often addressed with *in vivo* or *ex vivo* stone removal procedures before renal transplantation. Some authors opt for SWL/RIRS before transplantation [104, 105], while others favour *ex vivo* URS or pyelolithotomy immediately after donor nephrectomy [106–109]. The development of UTI is a significant concern in renal transplant recipients; single-use flexible uretero-renoscopes appear to reduce the risk of iatrogenic UTI during *ex vivo* URS [106]. A single-centre observational study (NCT05519150) is being conducted to evaluate the applicability and safety of kidney transplantation from donors with known nephrolithiasis.

#### Management of hyperparathyroidism

Persistence of hyperparathyroidism, mostly defined as parathyroid hormone levels at least two times above normal although no consensus has been reached, within 12 months of kidney transplantation is referred as post-transplant hyperparathyroidism. Active vitamin D or cinacalcet are two most employed medical treatment options for hyperparathyroidism among kidney transplant recipients. Total or subtotal parathyroidectomy may be indicated in patients with persistent hyperparathyroidism among transplant recipients with accompanying hypercalcemia; though the recommended timing of such procedure is unclear, some evidence suggests an advantage of surgery prior to transplantation [110, 111]. However, the efficiency of such a procedure in terms of allograft function or survival or patient survival has not been well-established [112] with potential adverse effect being early reversible decline in allograft function [113, 114]. A retrospective case–controlled study involving 38 participants has demonstrated statistically significant decline in allograft function in the first 5 days after parathyroidectomy procedure without any considerable difference in long-term follow-up [113]. A novel approach referred as ultrasound-guided microwave ablation has shown to be a safe and effective alternative to surgical parathyroidectomy [115]. An important consideration deciding between surgical or medical therapeutic alternatives for the management of post-transplant hyperparathyroidism is assessing the potential adverse events including surgical procedure-related risks and vitamin D or calcium supplementation-mediated potential hypercalcemia, hypercalciuria, nephrocalcinosis and nephrolithiasis risks.

Additionally, another important consideration is the kidney transplant candidates with secondary hyperparathyroidism and their therapeutic approach. Dietary interventions, conservative measures, cinacalcet and parathyroidectomy are potential therapeutic options for secondary hyperparathyroidism cases. Even though properly functioning kidney allograft after transplantation may hypothetically reverse CKD–bone mineral disorder and hyperparathyroidism, it is not always the case. A longitudinal follow-up study involving 911 adult patients with a mean follow-up period of 47 months and estimated GFR >30 mL/min/1.73 m^2^ has demonstrated 62% prevalence for persistent hyperparathyroidism after 1 year of transplantation with statistically significant association with death-censored graft survival after adjustment for multiple confounding factors (*P*-value = .009) [116]. Secondary or tertiary hyperparathyroidism have been linked to poor allograft function with superiority of pre-transplant parathyroidectomy over no therapy [117]. Nevertheless, there is no consensus on optimal therapeutic approach for the management of secondary hyperparathyroidism before kidney transplantation. A retrospective cohort study involving 334 kidney transplant recipients has demonstrated superiority of pre-transplant parathyroidectomy compared with cinacalcet therapy in terms of post-transplant serum calcium or parathyroid hormone levels (*P*-value = .003) without any considerable difference in terms of short or long-term allograft survival [118]. Another large-scale clinical study involving 5094 adult patients receiving treatment for secondary hyperparathyroidism with either cinacalcet (*n* = 4866) or parathyroidectomy (*n* = 228) has failed to demonstrate any difference in terms of delayed graft function, graft failure or death among groups. Nevertheless, the risk for tertiary hyperparathyroidism is significantly higher in patients who underwent maintenance dialysis over 3 years if they received cinacalcet therapy compared with parathyroidectomy [119]. Moreover, pre-transplant parathyroidectomy have shown to be superior in terms of calcium metabolism and allograft function compared with post-transplant parathyroidectomy [111, 120]. Therefore, we recommend management of secondary hyperparathyroidism either with pharmacotherapy or surgery with close follow-up of calcium metabolism and serum parathyroid hormone levels along with referral to surgery among refractory secondary hyperparathyroidism to cinacalcet. Moreover, we recommend planning of parathyroidectomy surgery prior to kidney transplantation when such therapeutic option has been chosen.

## CONCLUSION AND FUTURE DIRECTIONS

Nephrolithiasis after kidney transplantation is an under-investigated condition with limited evidence for prevalence, risk factors, outcomes and therapeutic approaches. Approximately 1%–2% of all kidney transplantations are thought to be affected. In contrast to the general adult population, nephrolithiasis in transplant recipients is more likely to be asymptomatic due to the denervation of donor kidney and ureter, contributing to a risk of underdiagnosis. Since the SFR of all active therapies is high, treatment choices can take into account the surgeon's and patient's preferences along with patient and stone features. Surprisingly, the limited evidence available does not indicate adverse effects of nephrolithiasis on allograft function. There is a clear need for large-scale, prospective clinical trials and observational studies for further evaluation of epidemiology and management of nephrolithiasis after kidney transplantation.
